# Supplementary material for: What you say and what I hear—Investigating differences in the perception of the severity of psychological and physical violence in intimate partner relationships
Source: PLoS One. 2021 Aug 18;16(8):e0255785. doi: 10.1371/journal.pone.0255785 (PMC8659990; doi:10.1371/journal.pone.0255785)
Supplement: Attachment — Submitted filename: What You Say and What I Hear _REVIEW 11 15 20.docx [file pone.0255785.s001.docx]

Thank you for the opportunity to review the manuscript entitled, *What You Say and What I Hear – Investigating Differences in the Perception of the Severity of Psychological and Physical Violence in Intimate Partner Relationships.* The stated purpose of the study was to assess potential perceptual differences in the communication of IPV in heterosexual romantic relationships. The authors stated, "This study focuses on measuring perceptual differences in the communication and perception of violence…To our knowledge these perceptual differences have not been explored…"

1. I applaud the authors for their pioneering work and found the study's aim to be relevant and important.
2. The manuscript would benefit for extensive editing to address several grammatical and spelling errors, beginning with the first sentence in the Introduction, which appears to have a typo, i.e. "A correction evaluation..". The authors may have intended to write "A correct evaluation". Elsewhere, under guidelines, we read, " If you are male rater, or are given evaluations of violence from a male *rather*, then consider increasing your sensitivity. I believe the authors meant to write "rater" . There are numerous other such examples throughout the manuscript and I encourage the authors to edit their work.
3. There are several sections that would benefit from being more concisely written, which would improve the work's readability. For example, the authors wrote, "According to our hypotheses, we expected to find"… This could have been reduced to "We hypothesized…"
4. I wonder if the relevance of the study would have been enhanced by adding raters who actually work in the criminal justice or legal systems…This is because they are likely to have experience which would inform the severity of their ratings.
5. The following sentence needs to be clarified: "*Gender PD exists if the seriousness ratings of violence depend on group belongingness of either the experiencer or the rater*".
6. The authors stated, "…*Thus, statements about psychological violence can be interpreted in more different ways across situations and individuals compared to physical violence. For instance, sarcasm and irony makes it more difficult for the receiver to fully comprehend the intention of the communicated statement. This leads to lower inter-rater reliability in the evaluation of seriousness of psychological violence*".

Please clarify the leap from comprehension of the intention of a communicated statement to inter-rater reliability.

7. I had hoped that the distinction among *Situational couple violence* ,*intimate*

*Terrorism, and violent resistance* would be included in the analysis. For example, were the raters informed of the distinctions among these concepts? Did the authors classify each narrative in these categories in order to determine which categories of violence were rated as higher in severity, either by the "experiencer" or by the rater?

8. Was sexual violence was rated as physical violence in this study. Research indicates that sexual violence adds layers of psychological trauma that raters, in this case, might rate as more severe, especially based on the gender of the victim/survivor.
9. The authors stated, "*First, the difficulty of communicating psychological violence may lead to a Calibration PD where psychological violence is perceived as less serious when it is communicated"*…

This is an example of where complementary articulation of a study's findings would be enhanced by adding a qualitative component. For example, by selecting a subsample of participants for interviews from which the authors would obtain more detail regarding their experiences of psychological violence.

10. The authors stated*,"… psychological violence, being an indirect reinforcer, is more difficult to communicate than physical violence… It is argued that this occurs because psychological violence works as an indirect reinforcer, meaning that psychological violence requires a learning process. This does not apply to physical violence because it is a direct reinforcer*…"

There is a great deal of literature that indicates social learning is involved for both perpetrators and victims of IPV-

11. I have no problem with the fact that non-heteronormative persons were excluded from this study. I am just not sure if the reason provided is sufficient, i.e., "*The screening of heterosexuality was conducted because we were interested in changing the gender in the collected texts of violence, and we wanted to make sure that this occurred in close relationships consisting of a man and a woman"*. Trans women, for example, have a deeply felt internalized sense of being a woman.
12. "*Upon completion, they received £2.50 for their participation*". Please explain how the authors arrived at this sum as an incentive and provide a reference. Under limitations, include a discussion of whether or not this small incentive resulted in the loss of so much data that out of 136 enrolled, the final sample was 71.
13. The authors stated, "*The final data comprised of 68 narrations about physical violence and 68 narrations about psychological violence. These narratives were collected from a total of 71 narrators, as some of narrators only produced one narrative and others produced two* ".

Were there specific instructions regarding how many narratives were needed or was there a space limitation?

14. From an ethical perspective, what steps were taken to mitigate participants' psychological distress related to recall or re-experiencing adverse events such as physical or psychological violence? Any debriefing? Referral for psychological support as needed?
15. "*In Phase 3: If the participants had not experienced any psychological or physical violence, they were instructed to describe a situation that was as close to this violence as possible*".

I am not certain that the experience of being slapped, pushed down some stairs, beaten severely or some other direct act of violence correlates with "a situation that was as close to violence as possible".

16. The authors stated, "…*This included narrations involving pregnancy (because it would not make sense to have a story with pregnant men) or when pronouns were missing (because gender could not be identified in these narratives*)…"

I am confused, here. I thought part of the analysis was done by gender. What happened to narratives for which gender could not be identified? Were they discarded?

17. The authors stated, "*An analysis of simple main effects showed that whereas there were no difference in severity ratings for the female narratives in the two versions, but a significant difference in how the male narratives were rated with the stories in the gender reversed condition rated as more severe than those in the original version. This suggests that when a man is exposed to the same physical violence as a woman, he is considerably less likely to be seen as a victim. This effect can not fully be explained by the fact that men possess greater physical strength and are therefore (independent of the evidence) less likely to be victimised by a woman, because this explanation does not account for the fact that the same effect is found for psychological violence*".

The question which comes to mind is, aren't men often considered psychologically stronger than women also? Based on neuroimaging studies involving the role of the amygdala in emotional regulation Please see:

Eippert F, Viet R, Weiskopf N, Birbaumer N, Anders S. Regulation of emotional responses elicited by threat-related stimuli. Human Brain Mapping. 2007;28:409–423. [[PMC free article](https://www.ncbi.nlm.nih.gov/pmc/articles/PMC6871321/)] [[PubMed](https://www.ncbi.nlm.nih.gov/pubmed/17133391)]

See also:

McRae, K. et al. The neural bases of distraction and reappraisal. J. Cogn. Neurosci. 22, 248–262 (2010).

18. The authors stated, "*One possibility is that psychological violence is harder to communicate because it is based on secondary reinforcement, which may lead to it being harder to detect by the receiver of the information*" I think if the authors provide a clearer definition of secondary reinforcement as they are using it, it would clarify their point. ..
19. Under Guidelines, regarding gender, the authors recommend increasing sensitivity but do not indicate how this would be done. Also, please check grammar and typos in this section as well as others.
20. I was concerned about the following wording: "*If you are male rater, or are given*

*evaluations of violence from a male rather* (Typo, please edit), then *consider increasing your sensitivity, compared to if you are female, or given evaluations from a female rater*.

Again, just recommending that someone increases their sensitivity does not constitute a *fait accompli*; it's more likely to evoke cognitive dissonance. Guidelines need to be accompanied by "how to" instructions.

21. I would omit the entire guidelines from this manuscript, as the intent was not to write a manual. Stick to the purpose of the study. Your topic and findings are important enough.
22. "*Guidelines to victims of violence- If you are communicating psychological violence, try to be very clear of whether you find this violence severe, or not. This message may not get across the person you are communicating it to, and it may also be viewed milder than what you feel that it actually was. If you are male, try to emphasize the severity of violence more, the violence made to you may not be taken at face value*.

There is so much involved in reporting violence as a victim and so many phases one must go through (See Transtheoretical model of change and IPV), and once the decision is made to report IPV, the reporting itself is traumatic; So, the latter quote can appear as if the authors are engaging in "Victim blaming". I am sure they are not, but rewording that paragraph is important.

Other items that need to be addressed:

- DOIs missing from references
- Lots of grammatical and spelling errors throughout.
- Sometimes, no spaces are left after periods
- Sometimes, extra spaces are left between paragraphs
- Keeping "person" and "voice" consistent in the writing would be helpful. The authors shift from first person plural to third person- and from active to passive voice throughout the manuscript.
